# Pet-Friendly for Whom? An Analysis of Pet Fees in Texas Rental Housing

**DOI:** 10.3389/fvets.2021.767149

**Published:** 2021-11-08

**Authors:** Jennifer W. Applebaum, Kevin Horecka, Lauren Loney, Taryn M. Graham

**Affiliations:** ^1^Department of Sociology and Criminology and Law, University of Florida, Gainesville, FL, United States; ^2^Department of Research, Austin Pets Alive!, Austin, TX, United States; ^3^Humane Society of the United States, Austin, TX, United States; ^4^Independent Researcher, Toronto, ON, Canada

**Keywords:** pet-friendly housing, housing inequality, pet ownership, companion animals, pets, housing, human-animal interaction, animal welfare

## Abstract

Previous studies have underscored the difficulty low-income pet owners often face when attempting to secure affordable rental housing. Further exacerbating this housing disparity are fees charged on top of normal monthly rent to pet owners in “pet-friendly” rental housing. In this study, we aggregated rental housing listings from the twenty most populous cities in Texas, USA from a popular online rental database. We paired the rental listings with census tract information from the American Community Survey in order to investigate economic and racial/ethnic patterns in the spatial distribution of the properties. We find that less expensive pet-friendly listings were more likely to have pet fees charged on top of rent than rental units that were more expensive. Additionally, when pet fee burden was defined as a function of average income by census tract, low-income communities and communities of color were more likely than higher income and predominantly White communities to pay disproportionately higher fees to keep pets in their homes. We also find patterns of spatial inequalities related to pet fee burden by a metric of income inequality by city. The burden of pet rental fees may contribute to both housing insecurity and companion animal relinquishment. We discuss these findings as they relate to inequalities in housing, with particular attention to marginalized and disadvantaged people with pets. We conclude with recommendations for policy and practice.

## Introduction

Pet ownership is very common in the United States: recent estimates suggest that ~60% of households in the U.S. contain at least one pet (1) and it is likely this number has increased with the popularity of pets during the COVID-19 pandemic (2). While most pet owners consider their pets to be family members (3–9), pets are legally considered to be property and are therefore not afforded the same legal protections as human family members (10, 11). Notably, pet ownership is not a protected status under the Fair Housing Act and therefore tenants are not protected from housing discrimination on the basis of having a pet in their family (12). Moreover, there are no federal regulations limiting the amount of pet fees (i.e., upfront, one-time, non-refundable fee), pet deposits (i.e. upfront, refundable fee, provided there is no damage), or pet rents (i.e., monthly, recurring, non-refundable fee, regardless of damage) a landlord can charge, since rental laws vary by state (13). In Texas, the setting of this study, pet fees, pet rents, or pet deposits are all legal and there is no cap on their amount, although industry best practice is to make security deposits “reasonable” (14).

Despite the popularity of pets within U.S. households, their increased adoption during the pandemic, and the evidence that living with a pet may be beneficial for human health and wellbeing (15, 16), the capacity to realize such benefits varies markedly based on the resources to which one has access (17). A decade ago, Herzog pointed to possible differences in people's capacities to “choose” pet ownership, stating that people who have the time, energy, and economic resources needed to care for a pet may be better able to keep them for extended periods (18). Indeed, there is evidence that, despite the fact that companionship and social support from pets may be most beneficial in times of stress and adversity, there are many structural barriers and larger social inequalities that stand in the way for disadvantaged and marginalized people to keep pets in their families and households (17). For example, housing issues are a commonly reported reason for animal relinquishment, particularly among low-income individuals (19, 20). Studies investigating the demographic patterns of pet ownership have found that White people are more likely to own pets than those from other racialized backgrounds (particularly Black individuals), homeowners are more likely to own pets than people who do not own a home, and that wealthier people are more likely to own dogs than people with access to fewer economic resources (1, 21). More than ever, there exists a need to consider the potential inequalities in capacities to keep and care for pets, which could be improved through better understanding and addressing access to affordable rental housing for pet owners.

Pet ownership is identified as a mechanism for housing insecurity among renters (11, 22, 23). Families with pets report feeling powerless and discriminated against when they search for rental housing (11). Pet-friendly housing is often perceived to be of poorer quality and located in neighborhoods deemed less desirable (11, 22). Even so, families with pets report paying higher rents and fees (11, 22, 24). Families also report staying put, as they do not want to lose more money by having to pay another pet fee they will never get back, should they move into another rental (11). The low turnover rate of pet-friendly housing is therefore likely in part due to the practice of charging pet fees as opposed to any feelings of housing security or satisfaction. Meanwhile, the ability for landlords to fill a pet-friendly vacancy fast is likely due to the limited availability of units that accept pets (25).

Given these challenges, an estimated 20% of owners have been found to keep their pets in rental units illegally (24), yet by doing so they could be faced with eviction, a bad referral, or other ramifications (12, 22, 23, 26, 27). The relationship between pet ownership and eviction has not yet been explored directly in the literature; however, research has shown that renters who face evictions are more likely to relocate to poorer and higher-crime neighborhoods compared to those who move voluntarily (28). Furthermore, evictions that go through the court system result in a public record with little mechanism for expungement, which can damage a tenant's credit record and thus harm their ability to find future rental housing (29). Depending on the market, tenants who stay longer in their units may be at risk of “renovictions”–where landlords evict a long-term tenant and renovate the property, raising rents beyond what the last occupant could have afforded (30).

### Housing Inequality and Insecurity

In the U.S., low-income residents are increasingly challenged to find available and affordable housing. Homelessness and housing insecurity are being described as their own “epidemic” in the last year, as millions of renters became behind on rent payments due to the COVID-19 pandemic (31). Since June 2020, the U.S. Census Bureau has conducted Housing Pulse Surveys at two-week intervals to assess household needs related to food and housing security, employment, and access to education, among other issues. In August 2021, 7.9 million households reported being behind on rental payments and 5.8 million households reported having no confidence in being able to pay rent in September. Over 3.5 million households reported that they were very likely or somewhat likely to leave their current home within the next 2 months due to eviction (32).

However, millions of families were experiencing housing insecurity long before the COVID-19 pandemic began in the U.S. Princeton University's Eviction Lab estimates that there are ~3.7 million eviction filings in the U.S. each year and a 2020 report found that in 2019, 20.4 million renters were housing cost burdened (33). A family is defined as housing cost burdened if they spend >30% of their monthly income on housing expenses, including utilities and pet rent. A family is severely housing cost burdened if they spend >50% of their monthly income on housing related expenses (34).

Researchers estimate that just under one-quarter of all rental households in the U.S., or just under 11 million households, are extremely low-income, meaning that they are living at or below the national poverty level or make <30% Area Median Income (34). According to the National Low-Income Housing Coalition, in the U.S. there are only 37 affordable and available units for every 100 extremely low-income renter families (34). The lack of affordable housing options for extremely low-income families is not a localized event. As of August 2021, there is not a single state or metropolitan area in the country with enough housing that is affordable to extremely low-income families (34).

In Texas, the situation is even more dire, with only 29 affordable and available units for every 100 extremely low-income families (34). Over 838,000 families across Texas are considered extremely low-income renters and 74% of those renters experienced severe housing cost burden in 2020 (35). Nearly every major metropolitan area in Texas has a severe shortage of affordable housing, making it one of the lowest ranking states in the country for affordable housing. For example, in 2020, in the Houston metropolitan area there were only 19 affordable and available units for every 100 extremely low-income families. The San Antonio and Dallas-Fort Worth metropolitan areas had only 38 and 21 affordable and available units for every 100 extremely low-income families, respectively. Ranking last among Texas cities analyzed in 2020, the Austin-Round Rock region had a distressingly low 14 affordable and available units for every 100 extremely low-income families (35). None of these federal or Texas specific statistics account for pet-friendly affordable housing and it is likely that the housing stock that is both affordable to low- and extremely-low income families and accepting of pets is even smaller.

Beyond lack of available and affordable housing, landlord-tenant laws in Texas are landlord-friendly. Texas boasts one of the highest late fees in the country (36) and landlords can refuse to pay for repairs if tenants are behind on rent payment. Without stronger tenant protections in place, millions of renters are one emergency away from not being able to pay rent on time and, “the threat of eviction provides an omnipresent signifier that, for poor renters, their tenure is a contingent one” (p. 3) (37).

Housing insecurity is not race-neutral. Across the U.S., people of color are more likely to be housing insecure than White individuals, and these disparities were only exacerbated by the COVID-19 pandemic (38). This fact is linked to the racialized history of oppression in the U.S., and the legacy of redlining [the historic practice of systematic divestment in communities of color, notably with respect to mortgage lending (39)] is still evident in residential segregation today (40). For example, several researchers have outlined the pathways by which historical redlining of neighborhoods of color by the U.S. government and continued discriminatory practices led to systematic neighborhood disadvantages that trickle down to educational disparities, public health concerns related to environmental hazards, concentrated poverty, higher disease prevalence, and earlier mortality [see (41)]. Not only do people of color in the U.S. face wealth and income disparities that certainly determine the type of housing they can afford and the environments in which they live, they also often face continued race-related housing discrimination that can determine where they can secure leases, regardless of their ability to afford them (41).

### The Current Study

In this study we analyze rental housing data in Texas, U.S., in order to understand the extent to which renting with pets may create an additional cost burden for renters. Further, considering the state of housing discrimination and segregation among communities of color (42), we investigate the extent to which these fees may be a barrier to housing security for low-income individuals and people of color whose families include pets. As discussed above, Texas is a salient case study for investigating these relationships due to the lack of affordable housing combined with policy that tends to favor landlords over tenants. We build here on findings from Rose and colleagues (43) in a county in North Carolina, who showed that pet-friendly rental housing was more likely to be available to renters in predominantly White neighborhoods, compared to communities of color. Our analysis focuses on the spatial distribution of rental housing, as related to economic and racial-ethnic aggregated information by census tract, that is advertised as “pet-friendly.” Finally, we explore whether within-city income inequality is related to inequality in pet rent burden.

#### Hypotheses

##### Presence of Pet Fees

1. The presence of pet rental fees will be negatively associated with income such that lower income communities will have higher incidences of pet rental fees than higher income communities.
2. The presence of pet rental fees will more frequently occur in communities of color, compared to communities that are predominantly White.

##### Burden of Pet Fees

3. The burden of pet fees, defined as a percentage of median census tract income, will be greater for communities of color, compared to communities that are predominantly White.

##### Pet Fees and Within-City Inequality

4. Cities with greater income inequality, as measured by the Gini index, will be more likely to have greater spatial inequality in pet fee burden. Specifically, cities with high income inequality will have evidence of geographically close census tracts with notable fee burden differences.
5. Within cities with higher Gini indices, there will be observable relationships (as measured by linear regression modeling) between pet fee burden and the proportion of residents of color by census tract, and this effect size will be proportional to the Gini Index in each city.

## Methods

### Data Sources

Data were collected on January 19, 2021 *via* apartments.com by examining the available apartments in each target city. The top 20 cities in Texas were identified via the 2019 census estimate of overall population. The base query, https://www.apartments.com/-tx/pet-friendly/ was used to identify housing which was pet friendly. Then, the needed information from each housing sample was extracted and joined with census tract data collated from http://www.justicemap.org/ which was primarily comprised of data from the 2014–2018 American Community Survey by the United States Census (Tables 1, 2).

**Table 1:** Lists the cities, their population, and the number of samples from each in this dataset.

**City**	**Samples**	**Population**
Dallas	700	1,345,047
Houston	700	2,320,268
Austin	700	964,254
San Antonio	697	1,532,233
Fort Worth	696	895,008
Irving	346	242,242
Plano	327	287,677
Arlington	308	398,123
El Paso	202	682,669
Garland	195	242,507
Corpus Christi	170	326,554
Frisco	165	200,490
Grand Prairie	154	194,614
Lubbock	140	258,862
McKinney	114	191,645
Pasadena	82	153,219
Killeen	76	149,103
Amarillo	63	199,924
Laredo	41	261,639
Brownsville	35	183,392

*Note that the number of samples was capped at the 700 top results for pet-friendly locations due to limitations in the pagination of the data source*.

**Table 2:** Lists the key attributes for each housing unit as well as associated census tract attributes which were used as covariates during analysis.

**Attribute Name**	**Description**	**Data Source**
Median Income (Census Tract)	Median income of the census tract region within which the residence resides	Census
Two Bedroom Square Footage	Square footage of a two bedroom residence in the given entity	apartments.com
Two Bedroom Monthly Rent	Monthly rent of a two bedroom residence in the given entity	apartments.com
Recurring Monthly Pet Rent	Fees assessed monthly for a single pet	apartments.com
One-Time Pet Fee	Fees assessed one-time on move-in for a single pet	apartments.com
Proportion of: • White non-Hispanic • Hispanic/Latinx • Black or African American non-Hispanic • Asian • Native American • Native Hawiian and other Pacific Islander • Some other race • Multiracial/two or more races (Census Tract)	Proportion of the census tract identified as given race	Census

*When housing units did not have available data corresponding to these attributes, they were excluded from the data set. This could, of course, systematically bias the data toward higher-income housing if one assumes such housing has the funds to maintain more accurate listings. However, this is a limitation which will be present in most available online samples of housing data*.

Note that for all analyses in this paper, the fees for dogs are evaluated. Although the data contains fees for both dogs and cats, 5811/5911 (~98.3%) samples contained identical cost values.

These data contain 5,911 total samples with 3,875 of those having some form of pet fee (65.6%).

#### Outlier Removal

As the primary dataset in question is sourced from values reported on a website, significant outliers are present which can dramatically skew the results of an analysis which is attempting to examine the “typical” relationships among factors. The exact cause of each outlier was not examined in detail, but common causes included issues in parsing the site, null/missing data, and potential typos in the data (i.e., a monthly pet rent of 12,000 is assumed to accidentally contain extra 0 s). In order to avoid experimenter bias in the evaluation of what constitutes an outlier, an Isolation Forest was applied to the data to eliminate outlier points (44). The contamination proportion was determined automatically as per the original paper on the method and found to be ~11.8% [i.e., 458/3,875 samples containing pet fees; (45)]. Note that outlier removal was only performed for the subset of the data containing pet fees as the data was complete for the no-pet-fee group.

### Measures

In order to assess the typical impact of a pet fee on a potential resident, a metric called “Burden” (B) is introduced. The Burden is calculated as follows:


$$\begin{array}{l} B=\frac{12R_{p}+F_{p}}{I} \end{array}$$

Where the following variable meanings hold:

- **B** - Burden, i.e., income-proportional financial burden of pet ownership for typical residents
- *R*_*p*_ - The “pet rent” (i.e., monthly recurring fee) for the residential entity in question
- *F*_*p*_ - The “pet fee” (i.e., one-time fee associated with initiation of lease) for the residential entity in question
- **I** - The median income in the census tract within which the residential entity in question resides.

Note that this Burden value effectively assumes an individual may move apartments as often as once a year. Although this is likely an overestimate, it can be used as a benchmark to compare impact within and across regions. The value “12” could be modified to represent regional averages of occupancy times to generate a more accurate measure of real-world costs; however, these data were not available for this analysis.

### Analytic Strategy and Key Statistics

Several key questions were addressed in the analyses of these data. First, whether or not pet fees were present at residences labeled “pet friendly” was examined. As the majority of the data are not normal and not correctable to normal via typical transformations (square root, log, boxcox, etc.), and ANOVA results in non-normal residuals, a non-parametric Kruskal-Wallis H-test is employed to examine whether group differences in income, population, and racial/ethnic makeup relate to the presence of pet fees.

For evaluations of the magnitude of Burden compared to proportion of population comprised of people of color and income, because a linear model results in non-normal residuals, we employ bootstrapping, sampling 10% of the data 1,000 times and forming a distribution of linear model parameters to estimate the overall model parameters.

Significance level is set at alpha = 0.05 for all tests.

## Results

### Presence of Pet Fees

A significant relationship between the price of a two-bedroom apartment and the presence of pet fees was observed (H = 24.21, *p* < 0.001) such that more expensive apartments were less likely to have pet fees. No significant relationship was observed between the presence of pet fees and the proportion of people of color within the population in a census tract (H = 2.32, *p* = 0.13). Similarly, no significant relationship was observed between the overall population in a census tract and the presence of pet fees (H = 0.944, p = 0.33). A significant relationship between income and the presence of pet fees such that higher income census tracts were less likely to have pet fees was observed (H = 5.40, *p* = 0.02).

### Relationships Between Burden and Communities of Color

**Figure 1:**
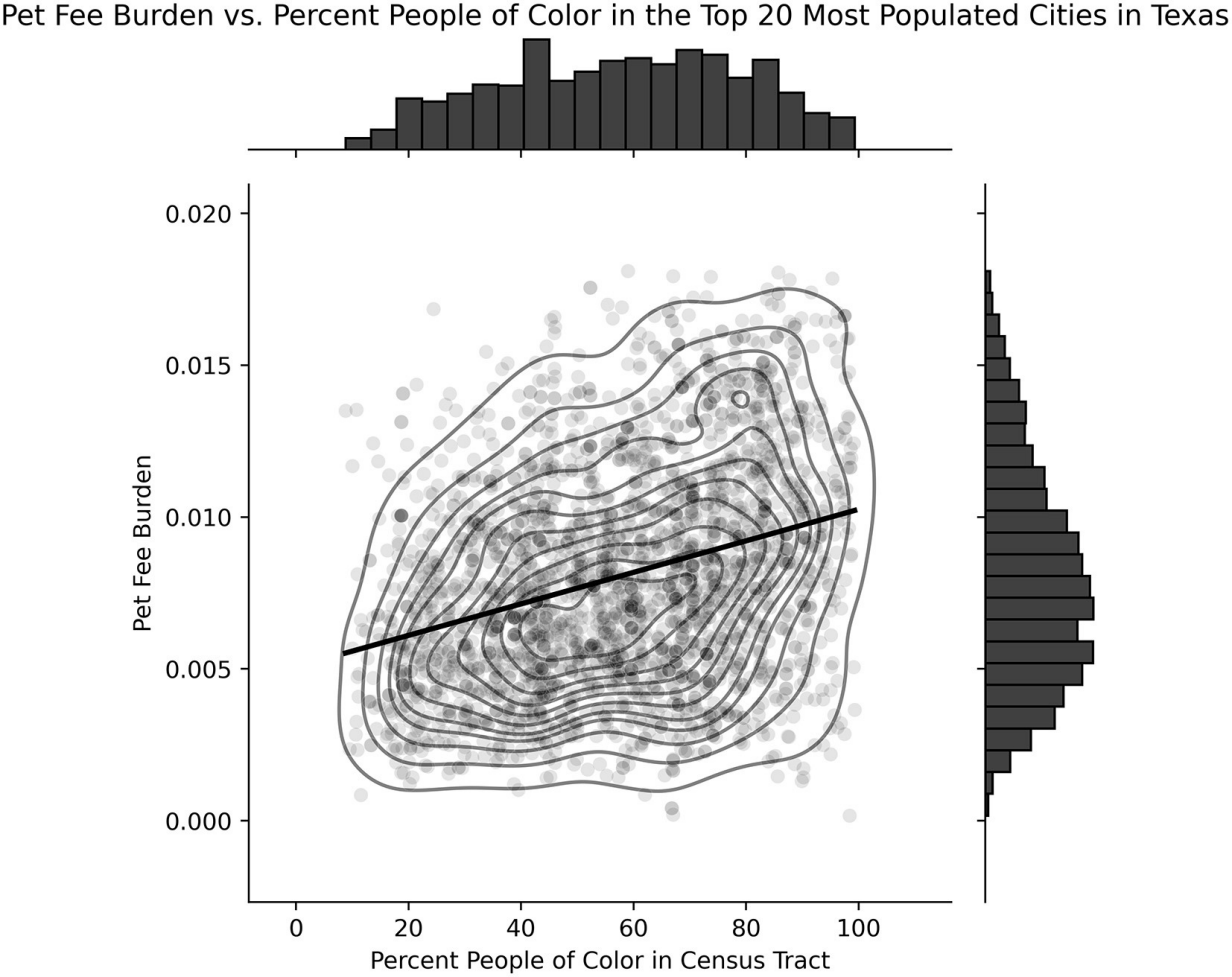
Relationship between pet fee burden and proportion of people of color by census tract. A significant positive relationship is present between the pet fee burden of a census tract's housing and the percent of people of color in that tract (*F* = 31.76, *R*^2^ = 0.10 and *P* < 0.001).

See Figure 1 for a visual representation of the relationship between pet fee burden and proportion people of color by census tract. Because the normality of the residuals of a linear model, bootstrapping with 1,000 iterations of 10% dataset samples was employed revealing a significant relationship between proportion of people of color in a census tract and pet burden such that census tracts with larger proportions of people of color were more likely to have a higher pet burden (F = 31.76, R^2^ = 0.10 and *P* < 0.001).

**Figure 2:**
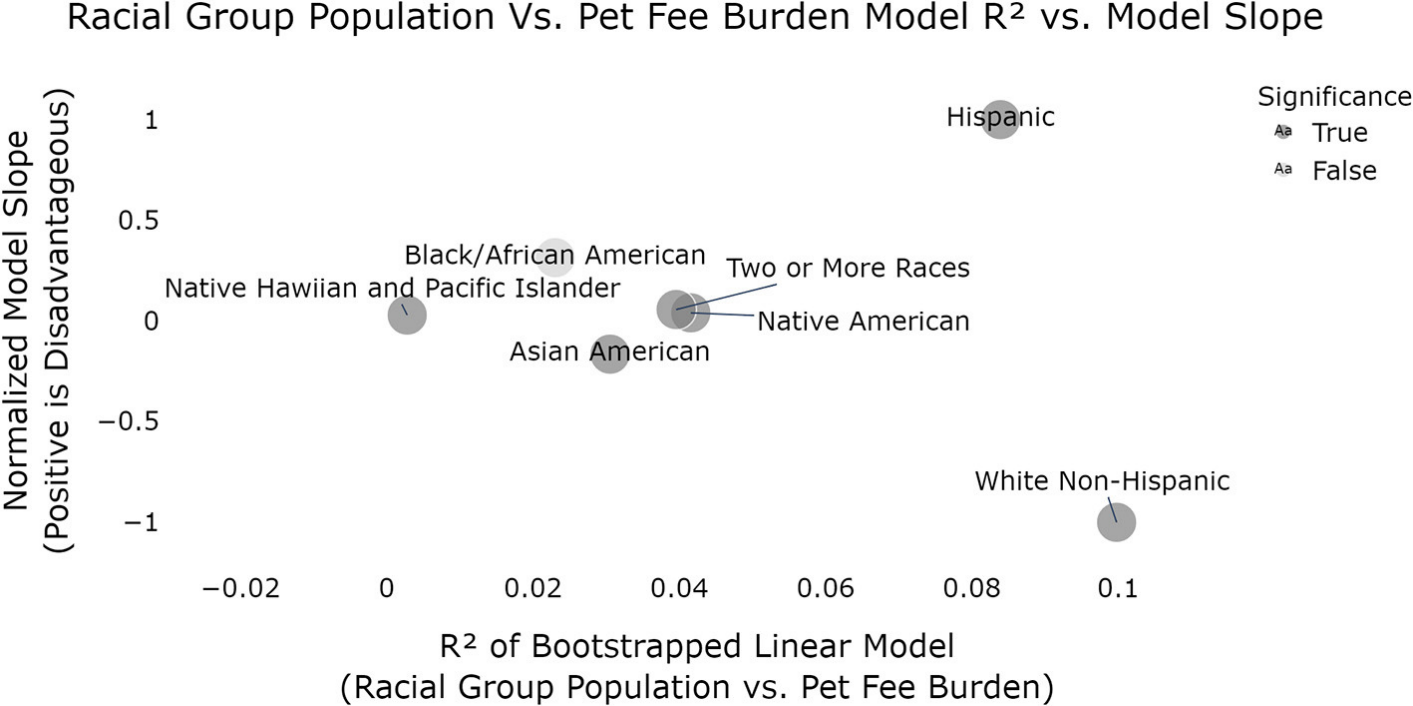
Relationship between model slope of proportion of people of color vs. pet fee burden by racial/ethnic groups and model R^2^ (dark gray is statistically significant models; *p* < 0.05). According to these models, White, Non-Hispanic populations are advantaged (in the form of lower pet fee burden) in relation to the racial makeup of their census tract while Hispanic/Latinx are the most disadvantaged. All models were significant except African Americans.

Finally, when we examine each racial group individually, we observe that for all groups except Black/African Americans, there is a significant relationship between the pet fee burden and the proportion of that group within the census tracts observed. Note, critically, although all of the significant models were fairly weak, they did not share consistent directionality. Hispanic/Latinx populations show positive slope (increased pet burden as proportion of Hispanic/Latinx population increases) while White and Asian groups show the opposite relationship (decreased pet burden as the proportion of White and Asian populations increases). See Figure 2 for visual representation of the relationship between model slope of proportion of people of color versus pet fee burden by racial/ethnic groups and model R^2^.

### Spatial Income Inequality and Pet Fee Burden Inequality

Finally, we examine the burden differences in extremely near geographic regions (<10 km) with the most extreme burdens (>1%) to examine the relationship between overall inequality in a city and the inequality as seen by Pet Fee Burdens.

**Figure 3:**
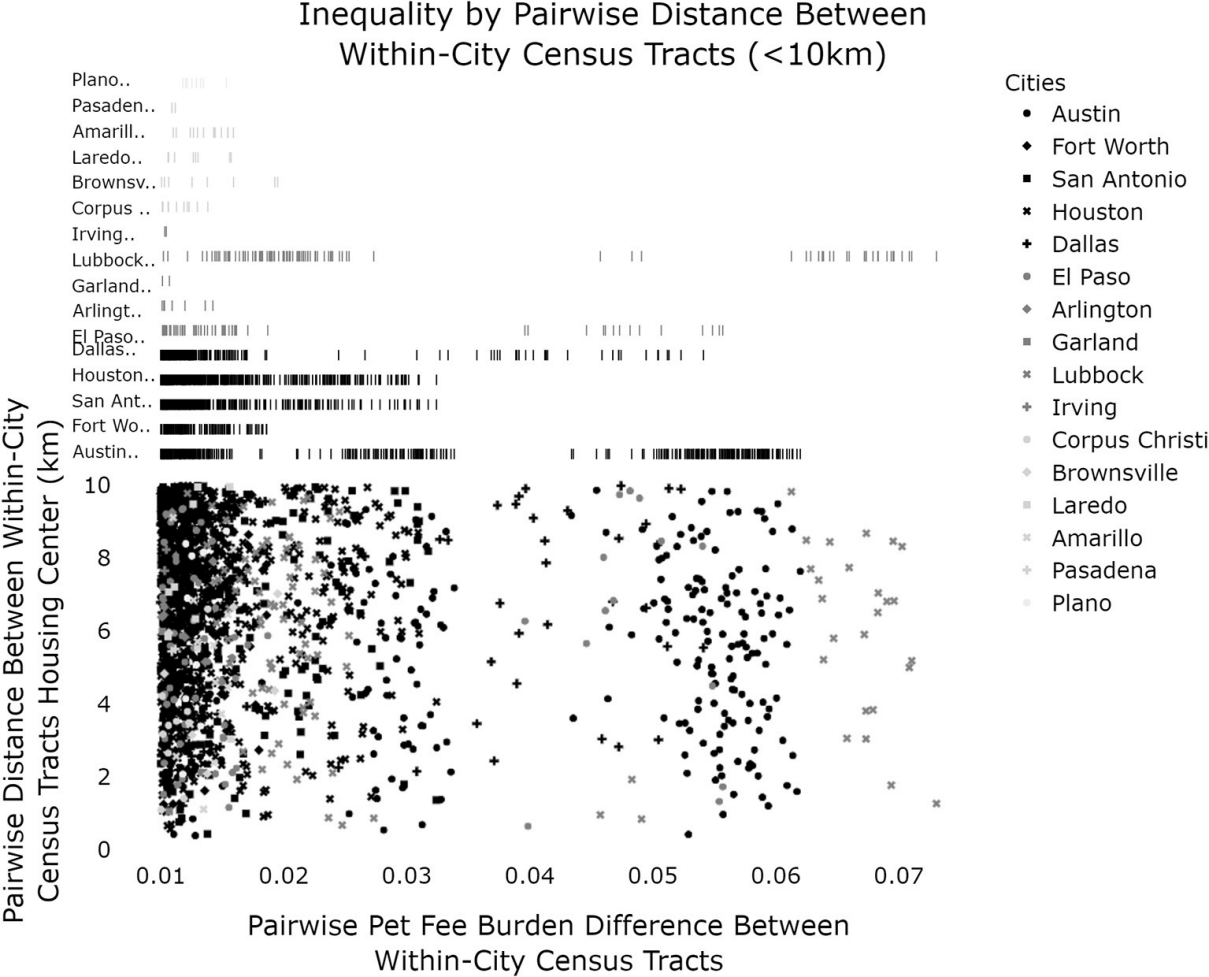
Relationship between relative pet fee burden difference by spatial distance by census tract. Pairs of census tracts within cities that are <10 km apart (from housing centroids) are compared and clear, city-wise stratification can be seen with Austin and Lubbock as the clear outliers.

**Figure 4:**
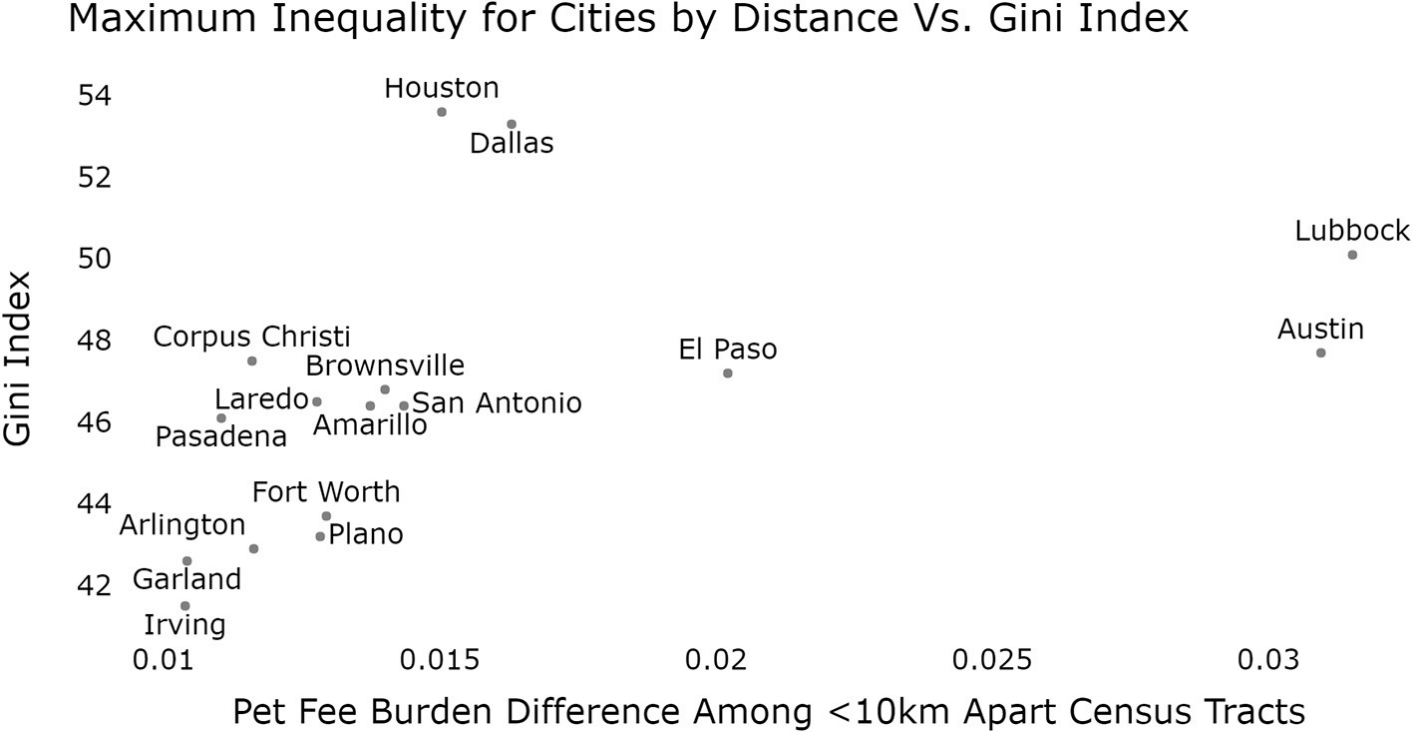
Relationship between Gini index and pet fee burden difference for census tracts <10 km apart, by city. Austin and Lubbock can be seen as clear outliers in their spatial pet fee burden difference with El Paso also possibly representing a deviation from the typical relationship.

In Figures 3, 4, we can see that Lubbock and Austin contain the most extreme pairwise disparities in Burden among close-together census regions. The points to the far right indicate six pairs of census tract regions which each are <10 km apart while having pet burden differences around 7%. A large band of similar pairwise census tracts can be seen for Austin around 6%. It is interesting to note that if we exclude the two extremes (Austin and Lubbock), there is a significant relationship between within-city pet fees disparity and the Gini Index of that city [a measure of economic inequality (46); F = 6.05, *p* = 0.03, R^2^ = 0.335]. This is confounded somewhat by the fact that both Gini Index and Pet Burden use Income as a factor component, however, were this to drive the relationship, strong outliers such as Lubbock and Austin would not be expected.

**Figure 5:**
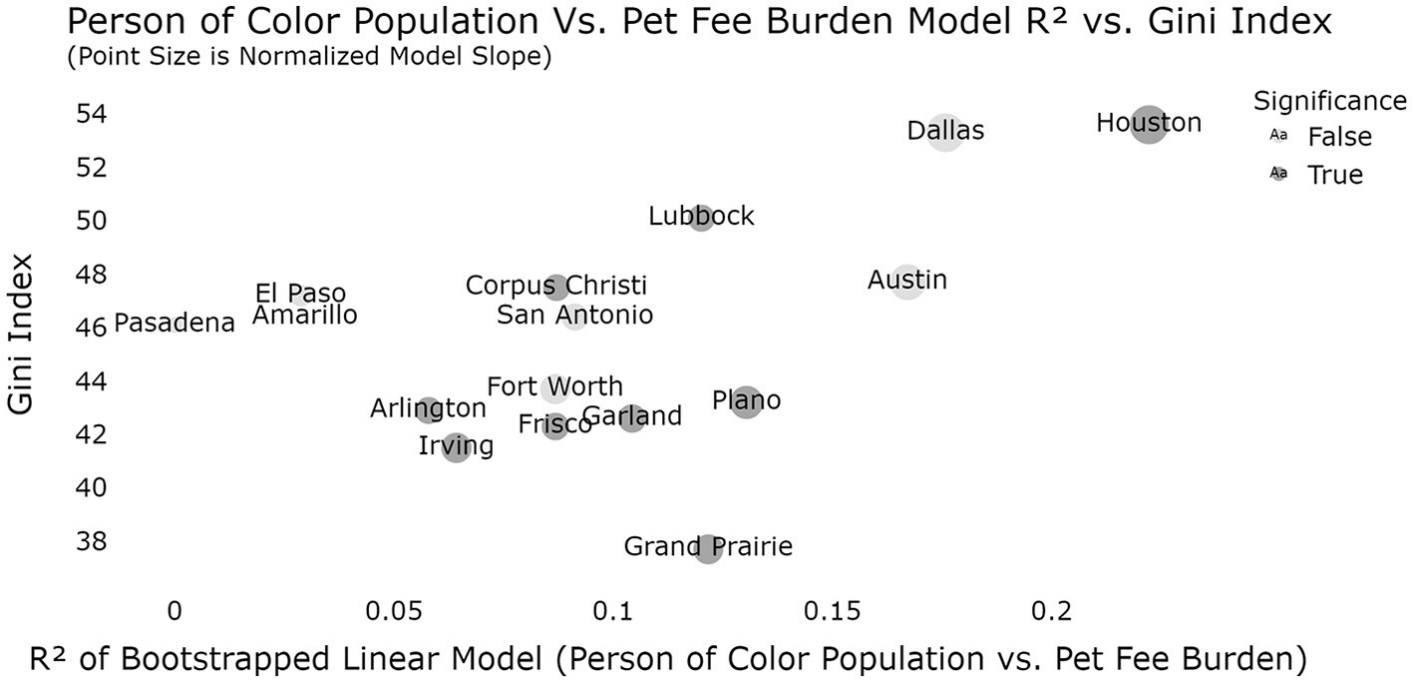
Relationship between proportion of people of color by Gini index by city with model effect size (dark gray is significant models; *p* < 0.05). Note that Houston is the city in which the evidence is strongest that inequality may be, in some way, related to the degree to which pet fee burdens can be predicted by racial makeup of the census tract.

**Figure 6:**
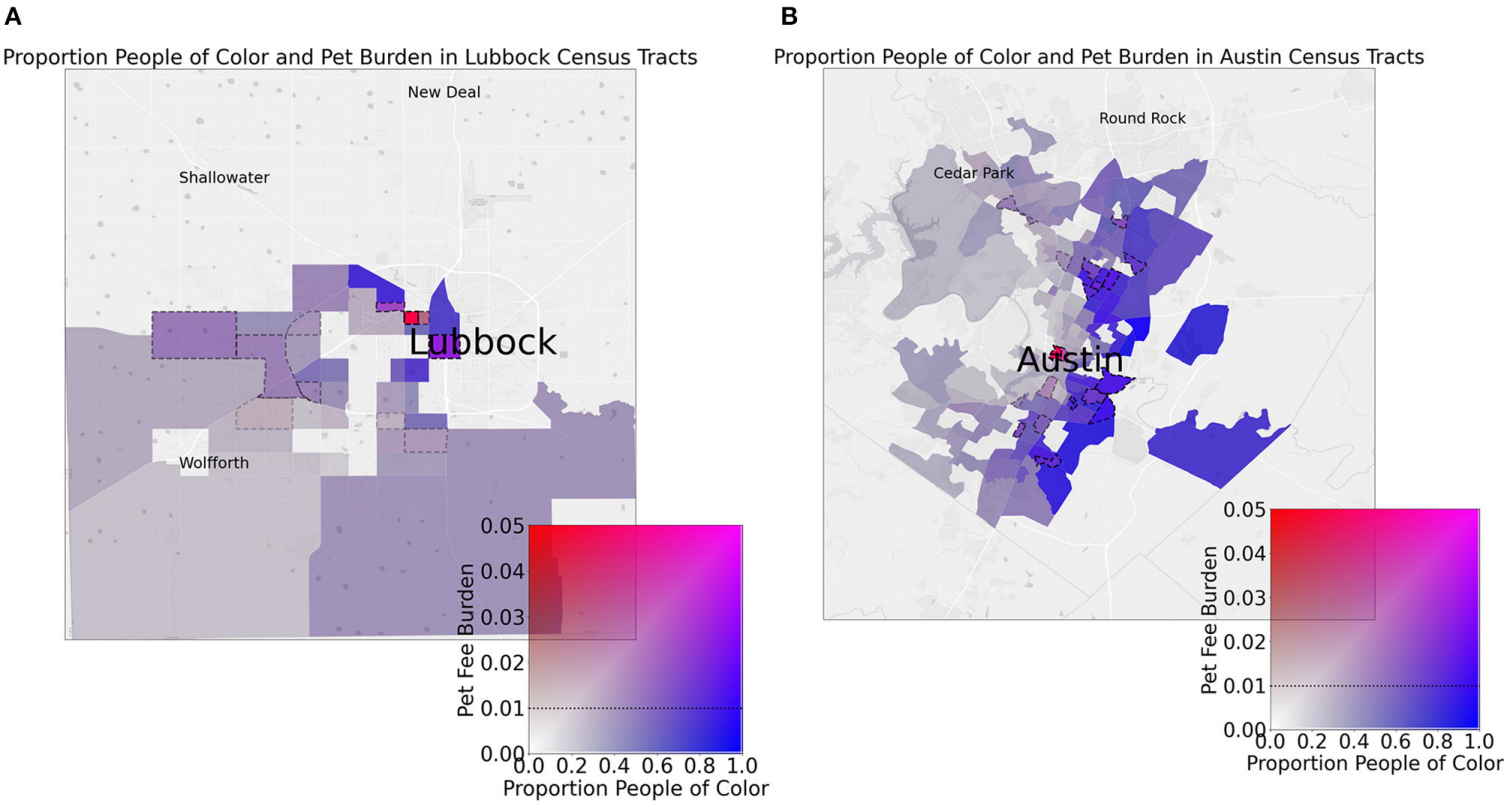
Maps display spatial relationships between census tracts' proportion of people of color (POC) and pet fee burden in Lubbock **(A)** and Austin **(B)**. Dotted lines around tracts indicate a 1% (0.01) or greater pet fee burden. Note that highly blue tracts indicate low burden POC areas while purple regions indicate high burden POC areas. Red indicates high burden, low POC areas while transparent regions have low POC and low burden. It is interesting to note red hotspots at city centers while dotted line regions are typical of a large proportion of high POC regions.

**Figure 7:**
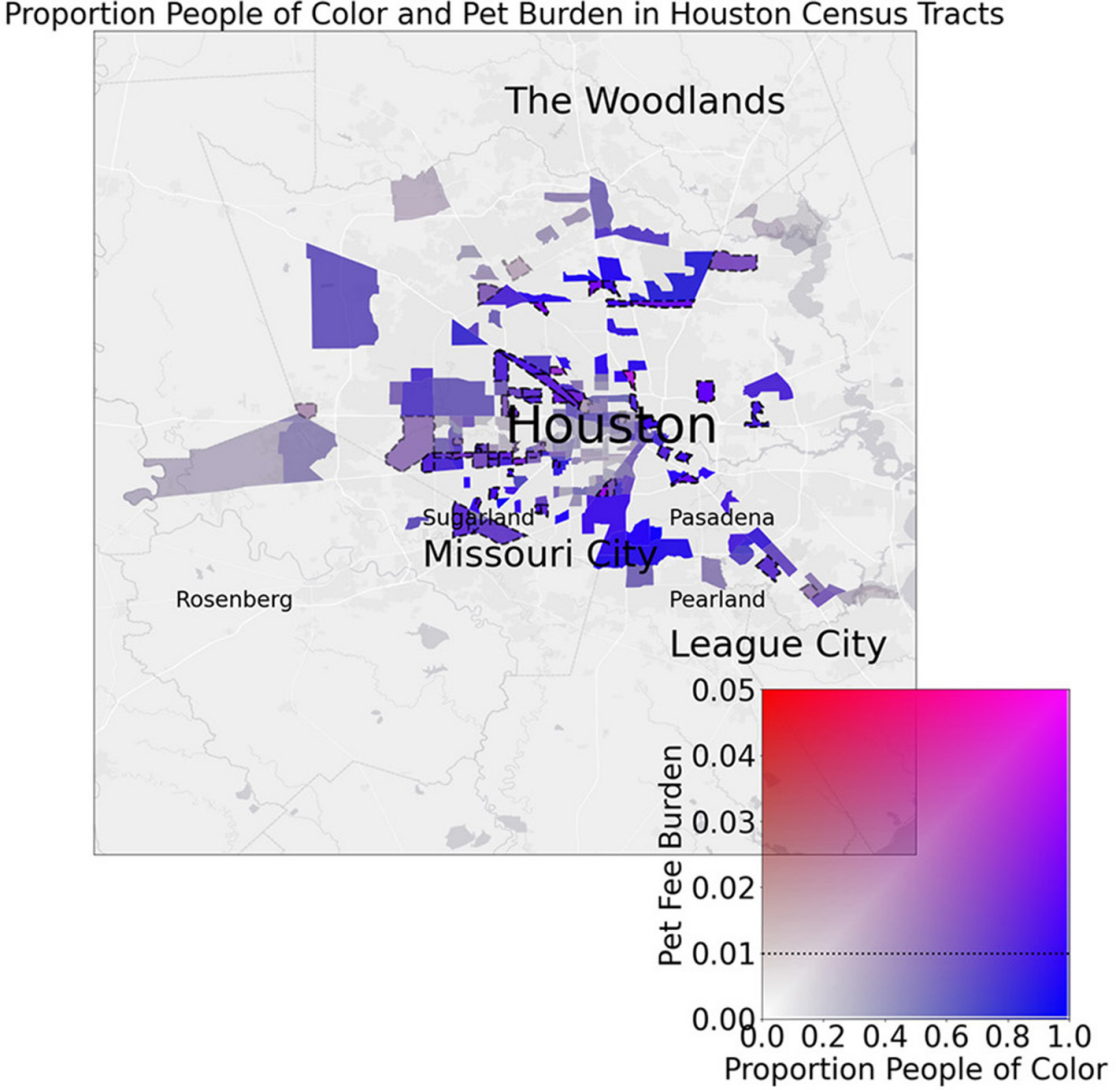
Map displays spatial relationships between census tracts' proportion of people of color (POC) and pet fee burden in Houston. Dotted lines around tracts indicate a 1% (0.01) or greater pet fee burden. Note that highly blue tracts indicate low burden POC areas while purple regions indicate high burden POC areas. Red indicates high burden, low POC areas while transparent regions have low POC and low burden.

Moreover, when the effect size of the bootstrapped linear models for each cities census tracts (proportion people of color vs. pet fee burden) is compared to the Gini index, Houston and Lubbock are observed to have both significant relationships and high R^2^ values (comparatively) in addition to high Gini indices (Figure 5). We can observe these cities directly to pinpoint clear regions of adjacent, unequal census tracts which might drive these effects (only Lubbock, Austin, and Houston are shown as they were the cities that were significant and/or outliers in the Gini index models) (Figures 6A,B, 7).

## Discussion

In this study we analyzed publicly available information about rental housing listings throughout the state of Texas in order to assess the additional cost burden placed on pet owners when renting with pets. Overall, our findings indicate that, within Texas, the costs associated with housing a family that includes a pet disproportionately harm populations that are already economically disadvantaged. Specifically, pet-friendly rental units come at a higher relative cost for low-income communities and communities of color. We elaborate on these findings in the following paragraphs.

First, we hypothesized that, among rental listings that advertise as pet-friendly, less expensive listings would more frequently include an additional fee to keep pets, compared to more expensive units. This hypothesis was supported. Specifically, we found that more expensive rental units were less likely to have pet fees, compared to less expensive units. This could imply that more expensive units already incorporate a “pet fee” into normal monthly rent, regardless of whether the tenant chooses to keep a pet or not. Additionally, pet-friendly listings within higher average income census tracts were less likely than lower average income census tracts to have pet fees on top of normal monthly rent. This finding builds upon previous literature showing that, overall, pet-friendly rental housing tends to be more expensive than housing that does not allow pets (11, 22, 27, 43). We also hypothesized that census tracts with higher populations of people of color, compared to census tracts that were predominantly White, would have higher incidences of pet fees. There was no evidence of this hypothesized relationship such that the presence of pet fees among pet-friendly housing did not appear to be related to the racial/ethnic makeup of the residents. This finding somewhat contradicts a study conducted by Rose and colleagues in North Carolina that showed that pet-friendly housing was less *available* in communities of color, vs. predominantly White communities (43). As we did not assess the relative availability of all pet-friendly housing due to data limitations (discussed further below), it is possible that the lack of relationship between race/ethnicity and presence of pet fees is at least in part due to the comparatively lower availability of pet-friendly housing, regardless of fees, in communities of color.

We defined pet fee burden as the total yearly pet-related cost (any monthly recurring fees plus any one-time fees such as those paid upon lease signing) by rental unit, divided by the median income of the corresponding census tract. This pet fee burden metric allowed us to examine the relative cost of keeping pets in homes as a function of a “typical” community member's yearly income. We hypothesized that communities of color would have greater pet fee burden among their pet-friendly rental listings, compared to communities that were predominantly White. Indeed, we found that overall, communities that were higher percentage White had lower pet fee burden, compared to communities that were higher percentage people of color. When examined by racial/ethnic group, as reported by the American Community Survey, we found that the pet fee burden was particularly pronounced for communities with higher populations of Latinx individuals. Notably, recent research has revealed the disproportionately high rent burden and concurrent barriers to access for rental assistance programs, overall, that Latinx individuals face in the U.S. (47). Taken together, Latinx individuals and families with pets may have a particularly difficult time obtaining housing. Conversely, the opposite relationship was found among both White and Asian communities: pet rent burden decreased as the communities had higher proportions of White or Asian residents. The relationship between pet rent burden and proportion of Black residents was positive in that higher percentages of Black residents indicated higher pet fee burden, though this finding should be interpreted with caution as it was not statistically significant. We suspect this non-significant relationship may be related to the limitations in our sample, which we discuss in more detail below.

We also hypothesized that cities with pronounced income inequality would be more likely to have greater inequality in pet fee burden among geographically close census tracts, and, given prior evidence that issues related to pets may exacerbate racial tensions [e.g., (48, 49)], this relationship would carry over to racial/ethnic disparities in pet rent burden. We find that Austin, Houston, and Lubbock show notable evidence of this relationship with respect to both income inequality and racial/ethnic disparities in pet ret burden. Specifically, Lubbock and Austin both had notable differences in pet fee burden among geographically close census tracts, and Lubbock, in particular, is among the highest in terms of overall income inequality (Gini index). However, the highest city-wide income inequality was observed in Houston and Dallas. Notably, Houston stood out in terms of pet fee burden disparities by race/ethnicity and was also represented by the highest Gini index. Houston's issues with racial housing segregation and income inequality are well-known; while Houston is the most ethnically diverse city in the U.S., it also has a long history of racial/ethnic and socioeconomic inequalities in housing and beyond (50). Houston is also especially vulnerable to severe weather events (particularly hurricanes) and it is likely that these events will grow more frequent and more severe due to climate change, which is predicted to further exacerbate racial and economic inequalities without strong policy intervention (51). There is evidence that pet ownership may be a risk factor for failure to evacuate during a disaster (52–54), and for those who do evacuate, pet owners can find it even more difficult to find rental housing following the disaster (27). Given this confluence of factors, disadvantaged and marginalized pet owners who live in Houston may be especially vulnerable to housing insecurity.

More than 15 years ago, a nationwide study on the lack of available pet-friendly housing in the U.S. concluded that opening properties up to pet owners makes “good business sense,” given the ability for landlords to charge more in rent and fees and given the benefits relative to risks (24). More recently, a 2021 report also emphasized the “economic opportunity” of pet-friendly housing for landlords (55), because pet-friendly vacancies are quicker to fill and tenants with pets tend to stay longer, thus keeping turnover costs low. Housing advertised as pet-friendly may attract more applicants and reduce tenant turnover (11, 22, 24) and thus be leveraged as a marketing tactic (56); nevertheless, it is essential to ask whether families renting with pets feel that they opted into the housing they are currently living in and can opt to stay or leave, rather than simply ending up there due to lack of choice.

Although all pet owners are affected by the limited rental housing options available to them, as our findings add to the body of literature, marginalized groups are particularly burdened, not only because of the discrimination they may already face—outside of pet ownership—in trying to find affordable housing (11, 43, 57), but also because of constrained financial resources and lack of reserve funds needed to pay pet fees, sometimes in addition to a security deposit (11). Paying a larger proportion of one's income on rent decreases the resources available for other necessities such as food, transportation, utilities, and healthcare, both for themselves and their pets. The landlord-friendly laws in the Texas rental market mean that families who are housing cost burdened are particularly susceptible to eviction, as evidenced by Garboden and Rosen, who interviewed landlords and property managers from Dallas, TX, Cleveland, OH, and Baltimore, MD regarding eviction practices (37). The authors classified Texas as the most “pro-business” of the states studied, noting that in Dallas, “if a tenant is late on their rent, they can be evicted, a unit turned over, and a new tenant housed by the beginning of the next month” (p. 8) (37). Furthermore, even when housing is advertised as pet-friendly, only pets of certain sizes, species, or breeds are permitted. Large dogs are especially hard to house, despite a lack of evidence suggesting that larger dogs are more problematic when housed (11, 22, 24).

Our findings point to the hypothesis that pet fees are yet another discriminatory practice that inevitably leads to poorer housing security and potentially increased evictions among already disadvantaged and marginalized populations. Additionally, considering previous research showing that people with pets may move to neighborhoods they deem “less desirable” in order to secure pet-friendly housing (11), it is possible that pet-related in-city residential mobility could contribute to gentrification, thus driving up housing costs in lower-income neighborhoods (58, 59). Evidence from the condominium market shows that “no pets” policies tend to drive up prices for units that do allow pets, and thus landlords may have a monetary incentive for keeping these policies in place (60). Overall, the problem with promoting pet-friendly housing as a strategy for landlords “to increase their bottom-line profits” (24) is that doing so disproportionately impacts marginalized groups. Rather than thinking about pet-friendly housing as an economic opportunity, we should consider ways to preserve families through fair housing practices.

### Limitations

This study is not without limitations. First, our sample consisted of publicly available data that was pulled from a popular online rental listing aggregator (apartments.com) and therefore is not representative of all available rental listings. Notably, the exclusion of listings from subsidized units likely biased our results in that the full extent of inequality was not evident. Likewise, it is possible that our non-significant result related to pet rent burden and proportion Black residents was related to this sample bias. Moreover, because this is an analysis of pet-friendly housing, it is possible that communities with higher proportion Black residents have less pet-friendly housing altogether, as was found in previous research (43). Because apartments.com will not display results past 700 listings per search criteria, we were unable to assess the proportion of overall rental stock that was advertised as pet-friendly (though this limitation only impacted the three largest cities: Houston, Dallas, and Austin).

We also acknowledge that using broad categorizations for racial/ethnic groups will inevitably remove some of the nuance related to inequalities in housing. For example, residents who were categorized in the American Community Survey as Latinx/Hispanic come from a variety of Latin American backgrounds and may identify as any race (e.g., White, Black, etc.). Thus, Latinx people are certainly not a monolith in terms of experiences of inequality related to housing and pets. The same goes for those who fall into the Asian census category, as Asian Americans have lineages from over 20 countries across Asia. Future research should consider the ways in which various racial and ethnic backgrounds may experience housing inequalities related to pets, beyond broad categorizations like those we have derived from the census here.

### Future Directions

Our findings point to several directions for future research. First, given our findings here, as well as those from previous research (11, 22, 56), it is likely that renting with pets may increase the risk of eviction. Future research should consider how pets may be a factor in the process of eviction, as well as the consequences of eviction for people with pets, and for the pets themselves. Relatedly, we are unaware of any research that systematically investigates the types of support available to families with pets when facing eviction. Second, considering our findings showing that pet fees are disproportionately unaffordable for low-income and marginalized individuals as a function of the area's median income, future research should interrogate whether the phenomenon of moving to lower-income neighborhoods in order to secure affordable housing that allows pets is widespread, thus potentially contributing to gentrification. Last, while Texas represents an important case study about housing inequality related to pets, future research should expand this study to explore national patterns. For example, do inequalities in pet-friendly housing differ by state, and does the political makeup of the state matter? These questions warrant investigation.

## Conclusion

In this study we assessed the cost burden of renting with pets in Texas. We found that higher cost “pet-friendly” rental units, which were generally within higher income communities, tended to be less likely to have pet fees, while less expensive units, which were generally in lower-income communities, tended to have additional pet fees on top of monthly rent. When we viewed pet rental fees as a proportion of the community's median income, we found that communities with higher proportions of residents who were not White tended to have higher relative pet rent burden, compared to communities that were predominantly (or entirely) White. In particular, pet fee burden was the most pronounced for communities with high proportions of Latinx residents. Finally, we found that there was a relationship between overall within-city income inequality and inequality in pet fee burden between nearby communities, as well as overall within-city income inequality and inequality in pet fee burden by proportion of residents who were people of color. Houston stood out as notable in terms of high overall income inequality, moderate spatial inequality in pet fee burden, and high racial/ethnic inequality in pet fee burden. We continue here with recommendations for policy and practice.

### Policy Implications

Given our findings suggesting that additional charges for pet ownership in rental housing disproportionately harm disadvantaged and marginalized pet owners, we continue here with several recommendations for housing policy. First, we strongly recommend against using the Texas Apartment Association's template Animal Addendum or other similarly punitive documents, and instead encourage landlords to adopt pet policies more reflective of the role that pets play in families. The Animal Addendum, for example, states that any single violation of the various rules as stated in the Animal Addendum or a single complaint by a neighbor can, at the sole discretion of the property manager, result in a written notice which will require a tenant to “immediately and permanently” remove the animal from the premises (61). Particularly disturbing is that the Animal Addendum allows a landlord to physically remove a pet when the tenant is not home following any rule violation or if a tenant allows their pet to “urinate or defecate where it is not allowed” (61).

Some states have much more tenant-protective, pet-prescriptive policies. In Kansas, for example, landlords can charge up to one month's rent for an unfurnished rental unit and are also allowed to charge an additional pet deposit of up to one-half of monthly rent (62). Similarly, in Nebraska, landlords can charge up to one-month's rent for a security deposit along with an additional one-quarter of a month's rent as pet deposit (63). Other states, like Arkansas, California, Maryland, Nevada, and Massachusetts, among others, simply place a maximum cap of security deposit that can be collected, regardless of how that deposit is designated. In these states, total deposits collected at the start of a tenancy range from 1 to 3 months, with some specifying whether or not the property is furnished (64). Both Montana and California prohibit non-refundable fees for any purpose, including fees for pets (65, 66).

Pet charges beyond the regular security deposit only add to the financial barriers that low-income tenants already face when trying to find housing. Past research has found that, in the rare instances in which pet-related damages do occur, security deposits are more than sufficient in most instances (24). Furthermore, there is no evidence that landlords charging for pet ownership are using this extra income to pay for any additional costs of maintaining rental properties that allow pets; to the contrary, housing advertised as pet-friendly is often perceived to be of poorer quality (11, 22). Knowing that tenants will go to extraordinary lengths to keep their pets, landlords in this case are merely capitalizing on the bond between pets and their people. If additional pet charges must be imposed, the amount should be a percentage of total monthly rent, be capped, and be made refundable, to incentivize good tenancy (11).

Finally, we recommend a blanket prohibition on “no pet” policies (with programs that reduce potential for pets to pose threats or nuisances when housed) throughout the rental housing market in the U.S. and to require all housing subsidized by local, state, or federal funding to be pet-friendly. For example, in August 2021, Illinois Governor Pritzker signed into law a landmark bill, S.B. 154, which requires housing providers receiving funding from the Illinois Affordable Housing Trust Fund to allow two cats or one dog up to 50 pounds and prevents landlords from prohibiting a dog based on its breed (67). While this Illinois bill signifies major progress in housing justice, it still allows for discrimination based on dog size and number of pets. Notably, most breed restrictions apply to dogs that are over 50 lbs. (e.g., Rottweilers, German Shepherds, pit bull-type dogs, etc.). In Ontario, Canada, there exists a province-wide ban on pit-bull type dogs; however, it is also the only province in Canada where it is illegal for landlords to reject housing applications based on pet ownership status. That said, the law is poorly enforced so it is not uncommon to see rental ads stipulating “no pets allowed” (56, 68). Once enacted, recommended policies must therefore be actively enforced and legal aid may also be needed, to help make tenants aware of their rights.

We acknowledge the above recommendations may be challenging to implement in practice. We suggest policymakers consider the full spectrum of possible interventions presented in this paper with our discussed recommendations as ideal solutions. We continue here with implications and recommendations for practice.

### Practice Implications

There is also a need to help promote a sense of security and positive community relations once families renting with pets are housed. Tenants living with a dog who barks incessantly when left alone, for example, may worry about neighbor complaints and getting evicted as a result (11, 69). Separation anxiety-related behaviors may be on the rise in the last year, as many dogs have become accustomed to being at home all or most of the day with their families during the pandemic (70, 71). Nonetheless, professional dog training services are expensive (72). One potential solution is for animal shelters to serve as resource hubs for issues related to pets in rental housing, for instance, offering a behavior helpline for tenants and landlords (11). Community outreach programs such as the Humane Society of United States Pets for Life program could also offer subsidized services including behavioral support, dog walking, and pet sitting to families renting with pets. Any such programs should address systemic issues and help build local capacity in marginalized communities so as to not cause further vulnerability or dependency. Finally, there is a need for neighborhoods to build safe and supportive outdoor spaces for dogs. Investments in sidewalks can motivate dog walking (73, 74) and access to dog parks can foster increased social interaction (75, 76), both of which can help keep dogs exercised and mentally stimulated so that they do not show problem behaviors inside.

## Data Availability Statement

The raw data supporting the conclusions of this article will be made available by the authors, without undue reservation.

## Ethics Statement

The studies involving human participants were reviewed and approved by University of Florida IRB. Written informed consent for participation was not required for this study in accordance with the national legislation and the institutional requirements.

## Author Contributions

JA, KH, and LL: conceptualization. KH: methodology and analysis. All authors writing, review, and editing.

## Funding

Research reported in this publication was supported by the National Center for Advancing Translational Sciences of the National Institutes of Health under University of Florida Clinical and Translational Science Awards TL1TR001428 and UL1TR001427.

## Author Disclaimer

The content is solely the responsibility of the authors and does not necessarily represent the official views of the National Institutes of Health.

## Conflict of Interest

The authors declare that the research was conducted in the absence of any commercial or financial relationships that could be construed as a potential conflict of interest.

## Publisher's Note

All claims expressed in this article are solely those of the authors and do not necessarily represent those of their affiliated organizations, or those of the publisher, the editors and the reviewers. Any product that may be evaluated in this article, or claim that may be made by its manufacturer, is not guaranteed or endorsed by the publisher.
